# Cytoprotective Activity of *p*-Terphenyl Polyketides and Flavuside B from Marine-Derived Fungi against Oxidative Stress in Neuro-2a Cells

**DOI:** 10.3390/molecules26123618

**Published:** 2021-06-13

**Authors:** Ekaterina A. Yurchenko, Ekaterina S. Menchinskaya, Evgeny A. Pislyagin, Ekaterina A. Chingizova, Elena V. Girich, Anton N. Yurchenko, Dmitry L. Aminin, Valery V. Mikhailov

**Affiliations:** 1 Laboratory of Bioassays and Mechanism of Action of Biologically Active Substances, G.B. Elyakov Pacific Institute of Bioorganic Chemistry, Prosp. 100 Let Vladivostoku 159, 690022 Vladivostok, Russia; ekaterinamenchinskaya@gmail.com (E.S.M.); pislyagin@hotmail.com (E.A.P.); martyyas@mail.ru (E.A.C.); daminin@piboc.dvo.ru (D.L.A.); 2 Laboratory of Chemistry of Microbial Metabolites, G.B. Elyakov Pacific Institute of Bioorganic Chemistry, Prosp. 100 Let Vladivostoku 159, 690022 Vladivostok, Russia; ev.girich@piboc.dvo.ru; 3 Department of Biomedical Science and Environmental Biology, Kaohsiung Medical University, No.100, Shin-Chuan 1st Road, Sanmin Dist., Kaohsiung City 80708, Taiwan; 4 Laboratory of Microbiology, G.B. Elyakov Pacific Institute of Bioorganic Chemistry, Prosp. 100 Let Vladivostoku 159, 690022 Vladivostok, Russia; mikhailov@piboc.dvo.ru

**Keywords:** marine fungi, secondary metabolites, cytoprotection, reactive oxygen species, paraquat, rotenone

## Abstract

The influence of *p*-terphenyl polyketides **1**–**3** from *Aspergillus candidus* KMM 4676 and cerebroside flavuside B (**4**) from *Penicillium islandicum* (=*Talaromyces islandicus*) against the effect of neurotoxins, rotenone and paraquat, on Neuro-2a cell viability by MTT and LDH release assays and intracellular ROS level, as well as DPPH radical scavenging activity, was investigated. Pre-incubation with compounds significantly diminished the ROS level in rotenone- and paraquat-treated cells. It was shown that the investigated polyketides **1**–**3** significantly increased the viability of rotenone- and paraquat-treated cells in two of the used assays but they affected only the viability of paraquat-treated cells in the LDH release assay. Flavuside B statistically increased the viability of paraquat-treated cells in both MTT and LDH release assays, however, it increased the viability of rotenone-treated cells in the LDH release assay. Structure–activity relationships for *p*-terphenyl derivatives, as well as possible mechanisms of cytoprotective action of all studied compounds, were discussed.

## 1. Introduction

Marine-derived fungi are a known source of antioxidants [1]. They produce secondary metabolites which can directly scavenge reactive oxygen species [2] as well as activate the Keap1/Nrf2/ARE antioxidant cellular machinery [3] and as a result upregulate antioxidant enzymes’ expression [4]. Both these direct and indirect natural antioxidants from marine-derived fungi protect the cells from oxidative stress (OS) induced damage. 

Paraquat (methylviologen, *N*,*N*-dimethyl-4,4′-dipyridynium dichloride) is the second most common herbicide in the world after glyphosate. Despite the protective measures applied, cases of illness and death from the toxic effects of paraquat are not uncommon [5]. The isoflavonoid rotenone is widely used in agricultural production as a pesticide. For a long time, it was believed that rotenone is not toxic to humans, however, in detailed studies, exposure to rotenone caused various toxic effects [6,7].

Paraquat induces the formation of reactive oxygen species (ROS) in cells as well as endoplasmic reticulum stress, apoptosis, mitochondrial damage, and inflammation, as evidenced by the regulation of various protein/signaling pathways [8]. Rotenone acts as a mitochondrial complex I inhibitor which resulted in the hyperproduction of ROS. In both cases, ROS overestimation leads to oxidative stress damage to DNA, lipid, and protein structures and mitochondrial dysfunction, as well as the activation of signaling associated with apoptosis.

**Figure 1:**
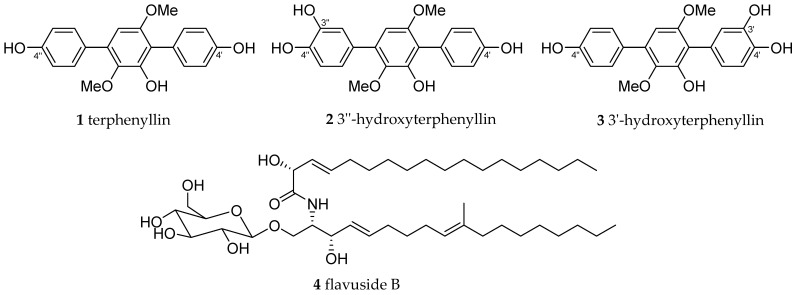
Chemical structure of investigated compounds.

During a large-scale study of low molecular weight secondary metabolites of marine fungi, it was found that a number of compounds protect murine neuroblastoma Neuro-2a cells against OS induced by 6-hydroxydopamine, paraquat, and/or rotenone [9,10,11,12,13]. As a part of this study, we investigated the influence of *p*-terphenyl polyketides **1**–**3** from *Aspergillus candidus* KMM 4676 [14,15] and cerebroside flavuside B (**4**) (Figure 1) from *Penicillium islandicum* (=*Talaromyces islandicus*) [16] against the effect of rotenone and paraquat on Neuro-2a cell viability and intracellular ROS level as well as DPPH radical scavenging activity. In this communication, we report the very first information available on these results.

## 2. Results

Polyketides **1** and **3** scavenged only 33.1% and 41.3% of DPPH radicals at concentrations of 100 µM and half-maximal effective concentrations (EC_50_) were not calculated (>100 µM). Compound **2** scavenged 64.6% of DPPH radicals at the same concentration and EC_50_ was calculated as 73.1 ± 3.1 µM. Scavenging activity of these substances was comparable to that of a standard antioxidant, ascorbic acid. Flavuside B (**4**) did not show any statistically significant radical scavenging effect in the DPPH test (Table 1).

Terphenyllin (**1**) showed a weak cytotoxic activity and a half-maximum decrease in Neuro-2a cell viability at a concentration of 101.5 ± 0.9 µM. Compounds **2** and **3**, as well as flavuside B (**4**), did not show any cytotoxic effect on Neuro-2a cell viability at concentrations up to 100 µM. In further experiments, all compounds were used at a non-toxic concentration of 10 µM.

**Figure 2:**
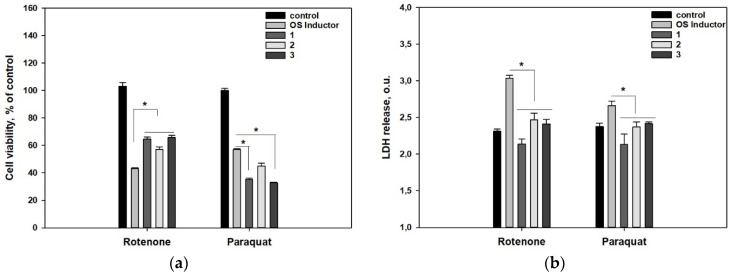
Influence of polyketides **1**–**3** on the viability estimated using MTT assay (**a**) and LDH release (**b**) in Neuro-2a cells treated with OS inductors, rotenone, and paraquat. Data are presented as mean ± standard error mean (SEM). * indicates significant differences (*p* ≤ 0.05). Differences between control and rotenone or paraquat were significant.

The influence of polyketides **1**–**3** on the viability and lactate dehydrogenase (LDH) release in rotenone and paraquat treated Neuro-2a cells is presented in Figure 2.

Rotenone at a concentration of 10 µM and paraquat at a concentration of 500 µM reduced cell viability by 59% and 42% compared to the control, respectively. All investigated polyketides **1**–**3** significantly increased the viability of rotenone-treated cells by 49.6%, 32.0%, and 52.3%, respectively. At the same time, compounds **1** and **3** decreased the viability of paraquat-treated cells more than paraquat only (Figure 2a).

Rotenone and paraquat significantly increased the LDH release from Neuro-2a cells by 31.3% and 11.9%, respectively. All investigated polyketides **1**–**3** diminished the LDH release from rotenone-treated cells by 29.6%, 18.7%, and 20.5%, and paraquat-treated cells by 19.9%, 10.9%, and 9.2%, respectively (Figure 2b).

**Figure 3:**
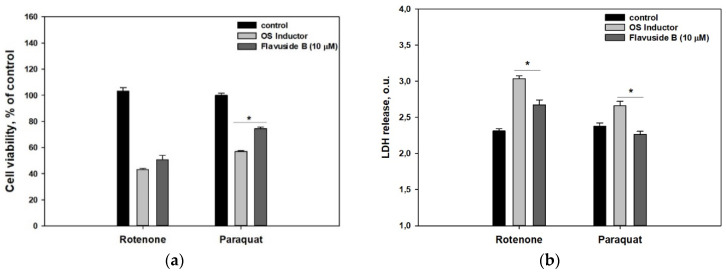
Influence of flavuside B (**4**) on the viability estimated using MTT assay (**a**) and LDH release (**b**) in Neuro-2a cells treated with OS inductors, rotenone, and paraquat. Data are presented as mean ± SEM. * indicates significant differences (*p* ≤ 0.05). Differences between control and rotenone or paraquat were significant.

The influence of flavuside B (**4**) on the viability and LDH release in paraquat- or rotenone-treated Neuro-2a cells is presented in Figure 3.

Flavuside B (**4**) increased the viability of paraquat-treated cells by 28.7% while its influence on rotenone-treated cell viability in the MTT test was not significant (Figure 3a). However, **4** significantly decreased LDH release both in rotenone- and paraquat-treated cells by 11.8% and 14.9%, respectively (Figure 3b). 

**Figure 4:**
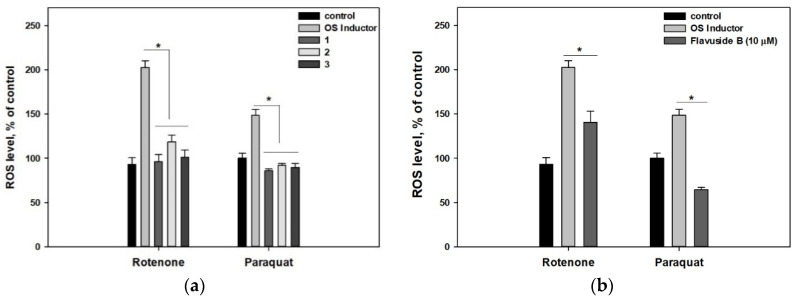
Influence of polyketides **1**–**3** (**a**) and flavuside B **4** (**b**) on ROS level in Neuro-2a cells treated with OS inductors, rotenone and paraquat. Data are presented as mean ± standard error mean (SEM). * indicates significant differences (*p* ≤ 0.05). Differences between control and rotenone or paraquat were significant.

The ROS level in cells incubated with rotenone for 1 h almost doubled in comparison with untreated cells (Figure 4). The ROS level in cells incubated with paraquat for 1 h, increased by 50% in comparison with untreated cells. Pre-incubation of cells with polyketides **1**–**3** significantly reduced ROS levels in rotenone- and paraquat-treated cells (Figure 4a).

Pre-incubation of rotenone-treated cells with **4** caused a reduction in the ROS level of 30.7% compared to rotenone-treated cells. At the same time, pre-incubation of paraquat-treated cells with flavuside B caused a significant diminishing of the ROS level by 56.8% compared to paraquat-treated cells (Figure 4b).

## 3. Discussion

Thus, terphenyllin (**1**) and their related compounds 3″-hydroxyterphenyllin (**2**) and 3′-hydroxyterphenyllin (**3**) showed significant anti-ROS activity in rotenone- and paraquat-treated Neuro-2a cells. Moreover, the compounds **1**–**3** significantly decreased the LDH release from these cells although this is the opposite of the results obtained in the MTT test. The MTT assay is a widely exploited approach to investigate the effect of compounds on cell viability but MTT reduction can be significantly affected by metabolic and energy perturbations, changes in the activity of oxidoreductases, endo-/exocytosis, and intracellular trafficking [17]. For this reason, using an alternative assay for cell death/viability (LDH release or trypan blue staining) detection is necessary [18]. Terphenyl derivatives **1**–**3** increased MTT reduction in rotenone-treated cells but they impaired MTT reduction in paraquat-treated cells. This could lead to the conclusion that **1**–**3** together with paraquat are more toxic to Neuro-2a cells, however, the data on the LDH release contradicts this conclusion. Pre-incubation of Neuro-2a cells with compounds **1**–**3** significantly decreased the LDH release in paraquat-treated cells, and the LDH release values in cells treated with compounds **1**–**3** and paraquat were not statistically less than in control cells. Thus, it can be assumed that a significant decrease in MTT reduction was caused by the peculiarities of cellular metabolism, and the studied *p*-terphenyl polyketides **1**–**3** actually have cytoprotective properties against rotenone- and paraquat-induced OS.

The presence of an OH group at the C-3 or C-3″ position in their chemical structure did not significantly affect the cytoprotective activity of *p*-terphenyl polyketides. At the same time, as it was shown earlier by us, another *p*-terphenyl polyketide candidusin A which possessed an ether bond between C-2 and C-2′ decreased the ROS level but did not affect cell viability in the MTT assay [13]. A free hydroxy group at C-2 in **3** instead of a bound hydroxy group in candidusin A allows for rotation around the C-1–C-1′ bond and significantly changes the stereochemistry of the molecule. Earlier, it was published that the modification of OH groups at C-4 and C-4″ as well as the presence of an OMe group at C-2′ in terphenyllin semi-synthetic derivatives resulted in a significant increase in the cytotoxicity of obtained compounds [19]. Probably, the presence of an OH group at the C-3 or C-3″ position, as well as a free hydroxy group at C-2 in the chemical structures of **1**–**3,** plays a key role in antioxidant and cytoprotective activities of *p*-terphenyl polyketides.

Flavuside B (**4**) statistically increased the viability of paraquat-treated Neuro-2a cells in the MTT assay and reduced the LDH release in these cells while the cytoprotective effect of **4** on rotenone-treated cells was not so significant. The positive effect of **4** on rotenone-treated cell viability was not detected by the MTT assay, while **4** statistically decreased the LDH release in these cells. The anti-ROS effect of **4** was incredibly significant in paraquat-treated cells and less against rotenone-induced OS. 

Obviously, differences between p-terphenyl polyketides (**1**–**3**) and flavuside B (**4**) activities are associated with the mechanism of the toxic action of paraquat and rotenone and, accordingly, the possible mechanism of the cytoprotective action of the compounds.

Rotenone is a direct inhibitor of mitochondrial complex I, also known as NADH:ubiquinone oxidoreductase (EC 7.1.1.2), which catalyzes the transfer of electrons from NADH to coenzyme Q10 and translocates protons across the inner mitochondrial membrane. Inhibition of mitochondrial complex I results in the overproduction of various ROS (peroxides and superoxides) that cause cell damage and death [20]. Paraquat causes the generation of intracellular free radicals via the reduction of the divalent paraquat ion (PQ2+) to the monovalent paraquat ion (PQ+) by NADPH-oxidation of mitochondrial complex I and reestablishment of a new redox reaction by PQ+, and induces OS in cells via the impairment of the redox cycling of glutathione and thioredoxin. Moreover, paraquat activates protein kinase delta, extracellular signal-regulated kinase 1/2, Jun N-terminal kinase, the caspase-3 signaling cascades, and other molecular signaling pathways [21].

Terphenyllin-related compounds have significant radical scavenging properties both in cell-free and cell-based assays and may act as direct antioxidants. In the case of rotenone, the direct antioxidant effect of *p*-terphenyls provides the scavenging of ROS and, as a result, increases cell viability measured in both MTT and LDH assays. At the same time, the direct antioxidant effect may not be enough to protect from paraquat toxicity, which causes more diverse toxic processes in cells, and cytoprotective influence was observed only in the LDH assay. However, this does not exclude the influence of **1**–**3** on antioxidant enzymes’ activity, especially as both **2** and **3** earlier reduced ROS formation and increased catalase activity in human intestine INT-407 cells treated with H_2_O_2_ [22].

Flavuside B did not show a radical scavenging effect in the DPPH test and increase the viability of paraquat-treated cells, and the diminishing of the ROS level may be caused by the indirect antioxidant influence of flavuside B. This is supported by the fact that flavuside B had no significant effect on the viability of rotenone-treated Neuro-2a cells.

Earlier flavuside B was founded only as an antimicrobial agent against *Staphylococcus aureus* and methicillin-resistant *S. aureus* [23]. Any cytoprotective activity of this cerebroside had not been reported, while other related cerebrosides showed anti-apoptotic and anti-inflammatory activities. Thus, longan cerebroside II from rhizomes of *Typhonium giganteum* protected PC12 cells from glutamate injury by downregulating the expression of caspase-9, caspase-3, and Bax, upregulating the expression of Bcl-2, and decreasing the level of cytosolic cytochrome c [24]. Cerebrosides from entomopathogenic fungus *Cordyceps militaris* inhibited the accumulation of pro-inflammatory iNOS protein and reduced the expression of COX-2 protein in LPS-stimulated RAW264.7 macrophages [25].

Thus, the cytoprotective effects of **1**–**4** on Neuro-2a cells treated with paraquat and rotenone were found, and the mechanism of their activity will be studied in detail in further research.

## 4. Materials and Methods

### 4.1. Compounds

The isolation of metabolites from marine fungi and their structural investigation have been reported previously [14,16]. Terphenyllin (**1**), 3″-hydroxyterphenyllin (**2**), and 3′-hydroxyterphenyllin (**3**) were isolated from ethyl acetate extract of the colonial ascidian-associated fungus *Aspergillus candidus* KMM 4676 (Shikotan Island, Northwest Pacific Ocean). Flavuside B was isolated from sediment-derived fungus *Penicillium islandicum* (depth 50 m, Aniva Bay, Sea of Okhotsk). The ascidian and sediment samples were collected with Sigsbee trawl during expeditions aboard research vessel “Akademik Oparin.”

The chemical structures of studied compounds are presented in Figure 1. Before the bioassays, all compounds were purified using earlier described HPLC procedures and their chemical purity was confirmed by HRESIMS [14,16]. All compounds were dissolved in DMSO (100%) at a concentration of 10 mM. These solutions were used to obtain the required concentration of compounds in the cell suspension so that the concentration of DMSO in the cell suspension did not exceed 1%.

### 4.2. DPPH Radical Scavenging Assay

The 2,2-diphenyl-1-picryl-hydrazyl-hydrate (DPPH) radical scavenging activity was tested as described previously [26]. The compounds were dissolved in MeOH, and the solutions (120 µL) were dispensed into wells of a 96-well microplate. The DPPH (Sigma-Aldrich, Germany) was dissolved in MeOH at a concentration of 7.5 × 10^−3^ M and the solution (30 μL) was added to each well. The concentrations of tested compounds in the mixtures were 10 and 100 μM. Pure MeOH was used as a negative control and ascorbic acid was used as a positive control. The mixtures were shaken and left for 30 min. The absorbance of the resulting solutions was measured at λ = 520 nm with a Multiscan FC plate reader (Thermo Scientific, Waltham, MA, USA). The results were presented as percent of the negative control (MeOH) data.

### 4.3. Neuro-2a Cell Culture

The cells of the mouse neuroblastoma cell line Neuro-2a (ATCC^®^ CCL-131™; American Type Culture Collection, Manassas, VA, USA) were cultured in DMEM medium (Biolot, St. Petersburg, Russia) containing 10% fetal bovine serum (Biolot, St. Petersburg, Russia) and 1% penicillin/streptomycin (Invitrogen, Carlsbad, CA, USA) at 37 °C in a humidified atmosphere with 5% (*v*/*v*) CO_2_. Initially, cells were incubated in cultural flasks until sub-confluent (~80%). For testing, Neuro-2a cells were seeded in 96-well plates and experiments were started after 24 h.

### 4.4. MTT Cell Viability Assay

The cells (1 × 10^4^ cells/well of a 96-well plate) were incubated with different concentrations of studied compounds for 24 h. After that, cell viability was determined by the MTT (3-(4,5-dimethylthiazol-2-yl)-2,5-diphenyltetrazolium bromide) method according to the manufacturer’s instructions (Sigma-Aldrich, St. Louis, MO, USA). The absorbance of the converted formazan was measured using a Multiskan FC microplate photometer (Thermo Scientific, USA) at λ = 570 nm. The results were presented as a percentage of control data.

### 4.5. Paraquat- and Rotenone-Induced Neurotoxicity

The cells (1 × 10^4^ cells/well of a 96-well plate) were treated with studied compounds at a concentration of 10 μM for 1 h. Then, 500 μM of paraquat (Sigma-Aldrich, St. Louis, MO, USA) or 10 μM of rotenone (Sigma-Aldrich, St. Louis, MO, USA) was added to the neuroblastoma cells and then incubated for 24 h. The cells incubated without paraquat/rotenone and compounds and with paraquat/rotenone alone were used as positive and negative controls, respectively. The viability of cells was measured after 24 h using the MTT method. The results were presented as a percentage of positive control data.

### 4.6. Lactate Dehydrogenase (LDH) Release Detection

The cells (1 × 10^4^ cells/well of a 96-well plate) were treated with studied compounds at a concentration of 10 μM for 1 h. Then, 500 μM of paraquat (Sigma-Aldrich, St. Louis, MO, USA) or 10 μM of rotenone (Sigma-Aldrich, St. Louis, MO, USA) was added to the neuroblastoma cells and the cells were incubated for 24 h. The cells incubated without paraquat/rotenone and compounds and with paraquat/rotenone alone were used as positive and negative controls, respectively. Then, the plate was centrifuged at 250× *g* for 10 min and 100 µL of supernatant from each well was transferred into the corresponding wells of an optically clear 96-well plate. An equal volume of the reaction mixture (100 μL) from LDH Cytotoxicity Assay Kit (Abcam, Cambridge, UK) was added to each well and incubated for up to 30 min at room temperature. The absorbance (optical units, o.u.) of all samples was measured at λ = 490 nm using a Multiskan FC microplate photometer (Thermo Scientific, Waltham, MA, USA).

### 4.7. Reactive Oxygen Species (ROS) Level Analysis

The cells (1 × 10^4^ cells/well of a 96-well plate) were incubated with compound solutions (10 μM) for 1 h. Then, paraquat (500 μM) or rotenone (10 μM) was added to the cell suspension to a resulting concentration for incubation for 1 h. Cells incubated without paraquat/rotenone and compounds and with paraquat/rotenone alone were used as positive and negative controls, respectively. The 20 μL of 2,7-dichlorodihydrofluorescein diacetate solution (H_2_DCFDA, Molecular Probes, Eugene, OR, USA) was added to each well (10 μM, final concentration) and the plate was incubated for an additional 10 min at 37 °C. The intensity of dichlorofluorescin fluorescence was measured with a PHERAstar FS plate reader (BMG Labtech, Ortenberg, Germany) at λ_ex_ = 485 nm and λ_em_ = 518 nm. The data were processed by MARS Data Analysis v. 3.01R2 (BMG Labtech, Ortenberg, Germany). The results were presented as a percentage of positive control data.

### 4.8. Data Evaluation

All data were obtained in three independent replicates and calculated values were expressed as mean ± standard error mean (SEM). A Student’s t-test was performed using SigmaPlot 14.0 (Systat Software Inc., San Jose, CA, USA) to determine statistical significance.

## Figures and Tables

**Table 1:** DPPH radical scavenging activity of investigated compounds.

Compound	% of Radical Scavenging at 100 µM	Compound	% of Radical Scavenging at 100 µM
**1**	33.1 ± 0.9	**3**	41.3 ± 2.1
**2**	64.6 ± 1.1	**4**	10.3 ± 5.9
Ascorbic acid	67.3 ± 3.4		

## Data Availability

Not applicable.

## Sample Availability

Samples of the compounds are not available from the authors.
